# Advanced Bio-Based Polymers for Astrocyte Cell Models

**DOI:** 10.3390/ma14133664

**Published:** 2021-06-30

**Authors:** Lidija Gradišnik, Roman Bošnjak, Tina Maver, Tomaž Velnar

**Affiliations:** 1 Institute of Biomedical Sciences, Faculty of Medicine, University of Maribor, Taborska Ulica 8, 2000 Maribor, Slovenia; lidija.gradisnik@um.si; 2 AMEU-ECM, Slovenska 17, 2000 Maribor, Slovenia; 3 Department of Neurosurgery, University Medical Centre Ljubljana, Zaloska 7, 1000 Ljubljana, Slovenia; roman.bosnjak@kclj.si; 4 Department of Pharmacology, Faculty of Medicine, University of Maribor, Taborska Ulica 8, 2000 Maribor, Slovenia

**Keywords:** astrocytes, tissue engineering, hydrogels

## Abstract

The development of in vitro neural tissue analogs is of great interest for many biomedical engineering applications, including the tissue engineering of neural interfaces, treatment of neurodegenerative diseases, and in vitro evaluation of cell–material interactions. Since astrocytes play a crucial role in the regenerative processes of the central nervous system, the development of biomaterials that interact favorably with astrocytes is of great research interest. The sources of human astrocytes, suitable natural biomaterials, guidance scaffolds, and ligand patterned surfaces are discussed in the article. New findings in this field are essential for the future treatment of spinal cord and brain injuries.

## 1. Introduction

Advances in medicine and cell biology have increased exponentially in recent years [1,2]. New technological developments and ever-changing opportunities have significantly influenced tissue engineering in many medical fields, including cell engineering and cell culture technology. Cells can be isolated from tissues and maintained in culture if suitable conditions exist for their growth and proliferation [3]. The development of cell and tissue culture is not new. It dates back to the early twentieth century. At that time, the pioneers of cell culture, Harrison and Carrel, developed the first techniques for isolating, and methods for maintaining, a cell culture, as well as techniques for studying cell physiology in a laboratory setting [3,4]. Under certain conditions, the isolated cells can be maintained outside the body, in the in vitro environment [1,2]. Among the first cell isolation techniques was the explantation method, in which cells migrate from a tissue sample and a tissue culture is formed [5,6]. Today, methods for isolating various plant, animal, and human tissues have become routine in research laboratories worldwide [6,7]. 

In vitro, these isolated cells are incorporated into so-called functional cell models, which are becoming increasingly important for the in vitro study of physiological and pathophysiological processes and are becoming an indispensable research tool in pharmacy and medicine to study cell transport and function, cell and drug interactions, drug bioavailability, carcinogenesis, and nutritional sciences [2,8,9,10]. There is no single cell line suitable for these models, so the isolation of various new types of cell lines from tissues is essential. An ideal cell model is one that most closely mimics in vivo conditions and consists of one or more cell lines. In experimental medicine, in the study of human pathophysiology, the utility and limitations of animal cells and transformed cell lines are well known [2,11]. However, the results obtained with these cells are not fully compatible and cannot be directly transferred to humans. Therefore, human-derived and non-transformed cells are preferable. In addition, experiments on cell cultures are becoming more common due to the declining trend for animal experiments and the associated lower costs [12,13]. Among the numerous isolated cell lines used for in vitro research and incorporated into functional cell models, central nervous system cells are an important research topic. Due to their abundance in the central nervous system, numerous functions, and variability in health and disease states, astrocytes and their functional cell models are frequently studied and are of great interest. Astrocytes are key cells in the central nervous system [14,15]. They are involved in many functions under physiological and pathological conditions. Primary cultures of astrocytes represent an important object for basic and translational neuroscience research, especially for in vitro cell models [16]. Astrocyte cultures for functional cell models are most commonly isolated from rodent brains, because they are easily accessible and grow rapidly [6,17]. Due to important differences between rodent and human astrocytes, culture of the latter is desirable [17,18].

Astrocytes have long been considered supporting and structural cells for neurons, mainly playing a passive role in the nervous system [19,20]. This view has gradually changed. Recent findings have highlighted their importance in complex and diverse roles, such as information processing in neural circuits and synaptic transmission. They make extensive contacts with blood vessels, are involved in directing and supporting neuronal migration, maintain the neural microenvironment, and serve as antigen-presenting cells [21,22]. 

Astrocytes play an important role in almost all central nervous system pathologies, including trauma, malignancies, and neurodegenerative disorders. Astrocytes are activated during inflammatory responses due to neuroinflammation and ischemia. Conversion of astrocytes to a reactive state is considered one of the most important pathological features in central nervous system pathology, not only in acute conditions, but also in chronic neurodegeneration. In in vitro models, these cells are used to study the process of neurodegeneration in Alzheimer’s and Parkinson’s disease, the pathogenesis of prion diseases, infections, trauma, and responses to toxins and drugs [23,24,25,26]. 

The main advantages of in vitro culture of human astrocytes include the possibility of biochemical analysis of individual cell types, a reduced cell complexity compared to the whole brain, the possibility of complete control of the cellular environment, individual cell imaging and electrophysiology, and co-culturing and manipulation of gene expression [27]. Adult astrocytes contain well-established connections and are better organized than newborn tissue, which is plastic and labile to stimuli. Consequently, astrocyte cultures obtained from humans can respond more reliably and help to elucidate the role of astrocytes in in vivo situations [28,29]. The cultured cells therefore represent an important new tool for in vitro studies. The availability of such a system allows the study of cell properties, biochemical aspects, and the potential of therapeutic candidates for traumatic and neurodegenerative diseases in a well-controlled environment [27]. 

## 2. Astrocytes and Tissue Engineering

**Figure 1:**
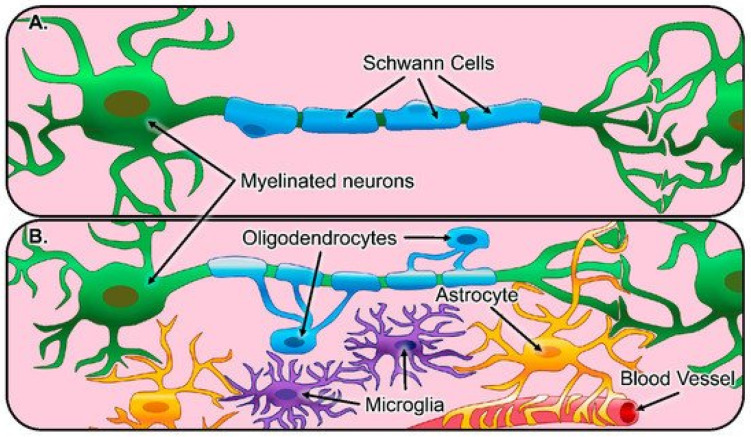
Illustration of glial cells and their function in the healthy nervous system. (**A**) The peripheral nervous system consists of Schwann cells (blue) that myelinate the axons of peripheral neurons (green). (**B**) The central nervous system consists of astrocytes (yellow) that regulate synaptic connections and comprise the blood-brain barrier, oligodendrocytes (blue) that myelinate axons of neurons, and microglia (purple), which act as resident innate immune cells [32].

Tissue engineering and biomaterial development represent a promising alternative to animal testing and provide an ideal opportunity to develop and test various biomaterials as scaffolds for purposes such as cell ingrowth and tissue repair [30,31]. Regeneration of the central nervous system is a particularly active area of research (Figure 1).

A variety of techniques have been used to create three-dimensional biomimetic scaffolds. In vitro studies have shown promising results with regeneration and repair [33,34]. Biomaterials have numerous functions, not only therapeutic, such as induction of axonal regeneration, neuroprotection, modulation of inflammation, and local release of therapeutics at the site of injury [35,36]. Moreover, biomaterial scaffolds can be engineered to facilitate and direct the spread of regenerating axons into white matter pathways [37,38,39]. Astrocytes play a crucial role in the regenerative processes of the central nervous system. They are the major class of glial cells in the central nervous system and are distributed throughout the brain and spinal cord. They are an essential cellular component of the brain, and in some areas, their number can reach up to 25% or 50% of the total volume, exceeding the number of neurons in humans [21,22]. They were considered structural and support cells for neurons for a long time, playing mainly passive roles in the nervous system, but recent findings have confirmed their multiple functions. Among the most important are synaptic transmission, information processing in neuronal circuits and functions, maintenance of the neuronal microenvironment, their role as antigen-presenting cells in modulating immune responses, and the control and support of neuronal migration during development [19,20,21,22,40]. According to their morphological appearance and distribution, astrocytes differ in their territorial organization and their physiological properties, including glutamate transporter and expression of proteins, the most important of which is a glial fibrillary acidic protein (GFAP) [15,22,41,42,43]. The brain microenvironment also plays an essential role in astrocyte function and structure. Not only the cells, neurons, and oligodendroglia are important, but also the composition of the extracellular environment in relation to the extracellular matrix. Its composition includes mainly proteoglycans, some laminin, tenascins, and hyaluronic acid. When culturing astrocytes for biomaterials research, these interactions are essential for obtaining the optimal cell phenotype.

Consequently, there are two significant challenges when considering astrocytes as biomaterials for brain research: (I) maintaining a non-activated state of astrocytes, with low GFAP expression; and (II) achieving a physiological morphology of the cells in culture. GFAP is a prototypical marker for the immunocytochemical identification of astrocytes because it is a reliable and sensitive marker [21,44,45,46]. Moreover, it is also crucial in biomaterial research and development of astrocyte cell models. To develop advanced and innovative biomaterials, the evaluation of astrocytes against biomaterials must include the analysis of many cytoskeletal proteins. GFAP is one of the most relevant, as it is known that biomaterials can affect the synthesis and expression of GFAP. Therefore, the quantification of this protein in cell culture is one of the most frequently studied parameters. Biomaterials that decrease GFAP expression are thought to favorably support neural regeneration. On the contrary, if the biomaterial increases GFAP expression, this implies a more reactive astrocyte phenotype that may not have a positive effect on regeneration [21,47]. This is important for biomaterial development because novel biomaterials need to stimulate astrocytes towards the phenotype that promotes axonal regeneration and neuronal survival [22,45].

A physiological morphology of astrocytes can be achieved by culturing the cells in a three-dimensional matrix that provides structural support and appropriate extracellular matrix factors, which allows the formation of the characteristic star-shaped morphology and low GFAP expression, which is an indicator of astrocyte activation [22,45,46].

To study the response of astrocytes to physiological and pathophysiological conditions, and to design biomaterials that interact favorably with astrocytes, in vitro experiments are performed in various cell models combined with different biomaterials. These can alter the phenotype of the cells, as discussed, and new ones are being developed to shift the phenotype towards low GFAP production. In these in vitro studies, astrocyte growth, proliferation, adhesion, morphological changes, migration, and gene and protein expression are determined [22,46]. The most commonly used biomaterials for astrocyte cell models include collagen gels, hyaluronic acid-based hydrogels, combinations of collagen and hyaluronic acid gels, gels composed of varying proportions of hyaluronic acid, collagen, and Matrigel, polymer scaffolds, and patterned substrates [22,42,48].

### 2.1. The Sources of Human Astrocytes and Their Importance for Cell Models in Biomaterial Research

Astrocytes are critical cells in the central nervous system [14,49]. They are involved in many vital functions under physiological and pathological conditions. Primary cultures of astrocytes represent an essential target for basic and translational neuroscience research, especially for in vitro cell models [16].

Primary cell cultures of astrocytes have been isolated from a variety of sources, usually rodent brains. Despite the abundance of experimental plants, there are significant differences between human and rodent astrocytes [50,51]. Human astrocytes differ from rodent cells in many ways. They are larger and more structurally complex and exhibit differences in calcium signaling. They also contact many more synapses than their rodent counterparts. In addition, humans and primates have astrocyte types that are not found in rodents [28,52]. These differences are the main reason for advancing and improving isolation methods and pushing forward studies on primary adult human astrocytes. On the other hand, human cells have the advantage of more accurately representing the environment of the central nervous system. Therefore, these cells are often used to study human central nervous system physiology and metabolic processes that would not otherwise be possible in vivo [53,54,55].

There have not been many reports on the isolation of human astrocytes [48,56]. The significant advantages of the in vitro culture of human astrocytes include the ability to perform biochemical analyses of individual identified cell types, reduced cell complexity (compared with whole-brain), the ability to fully control the cellular environment, the imaging and electrophysiology of individual cells, co-culturing, and manipulation of gene expression [27]. Mature astrocytes contain well-established connections and are more organized than newborn tissue, which is plastic and unstable in response to stimuli. Consequently, human astrocyte cultures respond more reliably and may help clarify the role of astrocytes in in vivo situations [28,29,57]. Therefore, it is more beneficial to study these cells separately under in vitro conditions [27,58,59].

Astrocytes can be isolated from different parts of neonatal or adult brains [28,60]. However, human tissue samples are usually obtained from neonatal brains. In rare cases, adult patients have also been designated as donors, mainly those who have undergone craniotomy for trauma, tumor, or epilepsy surgery, or have undergone surgery for various hemorrhages, such as arteriovenous malformations, intracerebral hematomas, and aneurysms. Postmortem specimens have also been collected [48,61]. When establishing cultures of human astrocytes, it is necessary to obtain a healthy part of the brain. If tumor cells are involved, tumor tissue can be used, as in various gliomas. When harvesting tissue, it is important to consider the pathology and the conditions under which the cells were harvested. For example, the brain substance surrounding the hematoma (i.e., the penumbra) is often not suitable for culture and isolation because it is necrotic or sub-vital, resulting in lower cell yield and quality [48,62]. 

Compared to adult astrocytes, neonatal astrocytes begin to show signs of ageing relatively late, after three to six months in culture. Initially, they grow and proliferate at a fast rate. Adult astrocytes have very limited proliferative activity in vitro and therefore do not remain in culture for long. They cannot be readily subcultured. These cultures are therefore of limited use. In addition, the differentiation of neonatal cells may be incomplete because they lack normal cell partners or differentiation signals [63]. They are also considered to be more activated than adult brain cells, which are mature [64]. This is particularly important when cell culture is used to study neurodegenerative diseases. The experimental results obtained from neonatal cells cannot be directly transferred to adult cells [65,66]. Therefore, adult brain-derived neuroglial cells form a useful and convenient model for experiments, as their pathophysiological mechanisms cannot be equally studied in neonatal culture. In recent years, isolation and culturing techniques have enabled better isolation capabilities for adult astrocytes [64,65]. In adult brains, tissue for isolation is much more readily available, both in quantity and frequency of collection, compared to neonatal brains. Neonatal brains can be obtained from fetuses, usually at 9 to 22 weeks of age, from elective abortions [67]. Furthermore, the timing of tissue collection is problematic and strict collaboration between the clinical department and the laboratory is necessary. Not all fetuses are suitable for isolation. Only brain-shaped fetuses collected after the surgical procedure of vacuum aspiration can be used. Tissue from fetuses that have undergone abortion after a medical procedure is not suitable, because the pharmaceutical agents used to kill the fetus can alter the viability of the cells and thus hinder the development of the primary culture [67,68]. On the other hand, adult tissue is readily available, as there are many more surgical procedures that can make tissue available for experimentation.

Transport to the cell laboratory is significant and the time and mode may vary. It is usually longer for neonatal brain samples collected during abortions. The transport time is typically less than two hours [69]. In adults, on the other hand, the tissue is usually more stable, as it is collected during resections and biopsies and reaches the laboratory much more quickly [70,71].

In the isolation and purification of astrocytes, one of the major limitations is that culture methods for mature astrocytes are not yet fully developed [72]. The technique developed by McCarthy and de Vellis in 1980, in which astrocytes were prepared from a neonatal rodent brain, has long served as a prototype for astrocyte isolation [73]. Much of our knowledge of astrocytes, synaptogenesis, and their role in neuronal survival comes from studies of these cells [74,75]. Although cultures isolated using this technique have increased our understanding of astrocytic function, they have a number of drawbacks. One is that these cultures select for populations of cells that express astrocytic markers but appear to have an immature or reactive phenotype. During the isolation process, only a small percentage of cells survive and proliferate, and the population is not prospective. The cells eventually stratify into two populations, the astrocytes and the oligodendrocytes. The latter grow on top of the astrocytes in culture and can be separated, leaving astrocytes in culture [72,76]. This technique is sophisticated and is used to select cells in neonatal animals that can survive and proliferate in vitro. It was originally developed for the isolation of rodent astrocytes and, with some modifications, has been applied to their human counterparts.

**Figure 2:**
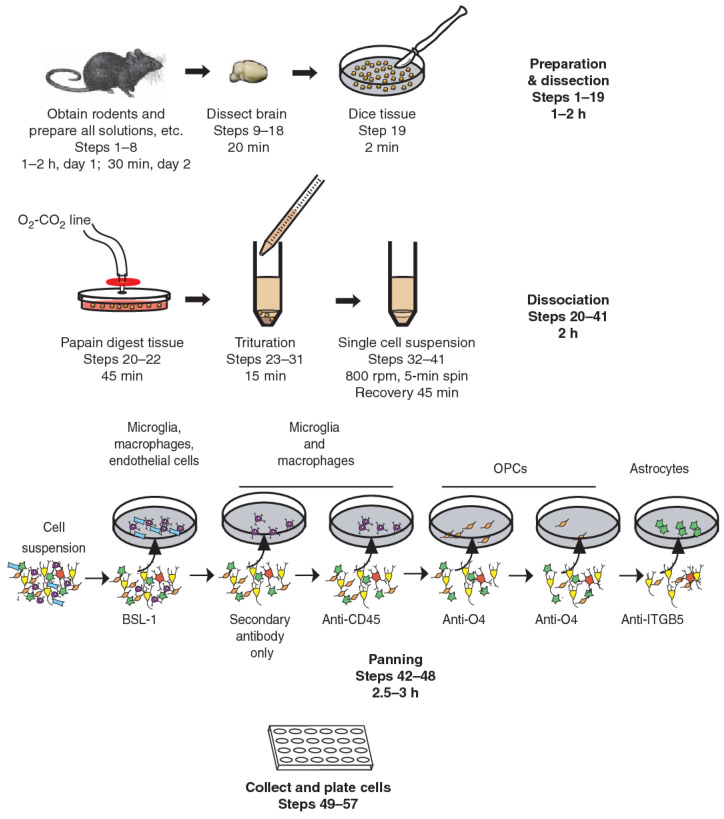
Immunopanning of astrocytes with anti-ITGB5 [80].

Immunopanning, on the other hand, is a new technique that allows the prospective isolation of astrocytes (Figure 2) [77]. This involves direct cell selection without multiple steps, allowing a representative population of astrocytes to be selected from an entire cell suspension. Immunopanning is a very gentle procedure and yields viable cells that can be cultured in serum-free medium containing heparin-binding EGF-like growth factor (HBEGF) at the end of the preparation. This factor is critical for the survival of astrocytes in culture, and in which they can be maintained for longer than two weeks. In contrast, astrocytes isolated by other techniques have a shorter lifespan in vitro [77,78,79].

Astrocytes isolated by immunopanning preserve their gene profiles and phenotypic characteristics. They promote neuronal survival and synapse formation and function in vitro [17]. Studies on immunopanned astrocytes revealed some of their important properties, such as findings on their phagocytic functions and the need for trophic support for their survival. These results indicate the importance of immunopanning-based purification of astrocytes for the study of their biology and function, and this isolation technique has been used for other species, not only rodents [72,76,77].

The progress made in recent decades in the isolation of astrocytes is also due to new technological achievements, laboratory techniques, and new surgical capabilities, including neuroendoscopic and neuronavigational methods and instruments. These allow tissue harvesting from different sites of the central nervous system, with minimal possible morbidity, and that is less invasive, more frequent, and with less tissue damage, which contributes to a higher cell yield when isolated in the laboratory [81,82]. There are numerous neurosurgical approaches used in clinical practice that provide a welcome source of both healthy and diseased brain tissue [83,84]. Over the past few decades, clinicians, researchers and patients have benefited from surgical techniques that optimize surgical outcomes, help limit the potential for neurologic morbidity, and increase the ability to obtain an ideal tissue sample, which forms the basis for successful cell isolation [80,83,85].

### 2.2. The Need for Tissue Engineering for Future Astrocyte Implantation

The biomaterials described in this article have been used in experimental applications. Spinal cord and brain injuries remain an important clinical problem for treatment, and functionally successful neuronal regeneration has not yet been achieved. Traumatic brain and spinal cord injury, stroke, and neurodegenerative diseases are a major case of morbidity and mortality worldwide and present a treatment challenge for clinicians and rehabilitation specialists [86,87]. Traumatic brain injury induces functional deficits due to axonal destruction and formation of cystic cavitations, scar tissue, and physical lacunae. In addition, reactive oxygen species are produced, leading to massive neuronal death, which worsens the course of secondary injury and may lead to disability and death [88,89]. In spinal cord injury, the mechanisms of neuronal damage are similar. In stroke, primary ischemic changes are followed by edema and altered vascular permeability, resulting in secondary brain injury, which further worsens the clinical condition and prognosis of patients. In such insults, not only neurons are affected, but also many other cells in the brain and spinal cord, such as astrocytes, microglia, oligodendrocytes, endothelial cells, and pericytes, which enter these pathological circuits [90,91]. As the central nervous system has a limited capacity to counteract the damage and dysfunction of axonal pathways and replace the lost neurons, these diseases often result in permanent neurological deficits [86,92,93]. Many attempts have been made to limit the extent of neuronal damage and to promote the recovery of the damaged brain and spinal cord areas, including limiting penumbra and promoting the regeneration of central nervous system cells. In in vitro research using biomaterials, cell-based approaches have been widely used in attempts to overcome the effects of glial scarring and replenish the lost cells, mainly neurons. The idea of bioengineering is production of cell carriers for implantation of axon growth promoting glia, and for supportive integration with host cells [88,91,94].

**Figure 3:**
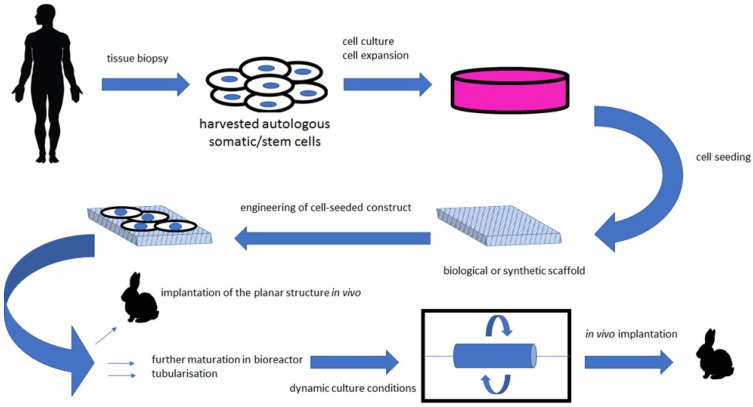
Bioengineered scaffolds as substitutes for grafts [98].

During the development of the central nervous system, neuronal migration and axonal expansion occur along corridors formed by other cells, particularly astrocytes [95]. These pathways or corridors are called living scaffolds. In regenerative medicine of the central nervous system, the goal is to simulate such scaffolds with artificial implants made of tissue-engineered biomaterials populated by one or more specific cell types, to promote neuron regeneration, enable targeted reconstruction, replace neural circuitry, and limit glial scar formation [96]. Such living scaffolds are constructed in vitro and can be implanted in vivo (Figure 3) to present cell adhesion molecules and neurotrophic and chemotactic signals that actively regulate neural migration and axonal growth during regenerative processes [95,97].

A variety of bioengineered scaffolds have been developed to promote axonal regeneration in damaged neural tissue. An ideal scaffold for such purposes does not yet exist. Among the most popular materials in bioengineering are collagens [87,94]. Biomaterials made from collagen have numerous advantages over many synthetic polymers, including having stability, non-toxic degradation products, biocompatibility, and the induction of minimal foreign body reaction [99].

Glial cells contained within engineered living scaffolds modulate numerous developmental mechanisms in the brain. Such tissue-engineered scaffolds often contain astrocytes, being the most numerous cells in the central nervous system. For example, hydrogel scaffolds may be coated with an extracellular collagen matrix and populated with astrocytes. Such scaffolds induce astrocytes to grow and orient into dense three-dimensional bundles of bipolar, longitudinally aligned projections. These aligned astrocyte networks provide a favorable substrate for neuronal attachment and neurite outgrowth. Moreover, these bio-manufactured scaffolds maintain their integrity and orientation even when detached from hydrogels, making them suitable for implantation into the central nervous system [92,95,100,101,102]. The neuroanatomical properties and potential of regenerative mechanisms may lead to a new class of engineered glial-based living scaffolds that can guide and promote the growth and expansion of immature neurons during migration and aid in axonal pathfinding through otherwise non-permissive environments. This can potentially mitigate the effects of neuronal degeneration that are so common in central nervous system injury and disease. So far, living scaffolds are only experimental and limited to use in animal research. A biomimetic, self-assembling peptide hydrogel has been tested in rats as stabilizing scaffolds and a vehicle for grafted cells following brain and spinal cord injury. They have been shown to be a suitable cell and drug delivery system in the injured central nervous system [95,97,103,104].

Biotechnological bridging materials include animal collagen, not just hydrogel. Experiments with glial cells and neurons have been performed on microstructured porcine collagen scaffolds. These contain densely packed and highly oriented channels that form a tri-structure that facilitates cell attachment, proliferation, and migration, making them suitable for tissue culture. Such biocompatible scaffolds that promote glial cell attachment and migration will be essential for future repair strategies for injured neural tissue [88,90,101].

Astrocytes are also known to be an essential component of the blood–brain barrier. They play an important role in its maintenance and repair, regulate amino acid, ion, and water homeostasis, and produce proteins to reinforce the blood–brain barrier [92,100]. Many in vitro models of the human blood–brain barrier integrate astrocytes and combine them with other cells, such as endothelial cells. Tight junctions of endothelial cells are essential in the blood–brain barrier models, indicating that these in vitro conditions are suitable for establishing important features of astrocyte and endothelial cell functions in the brain. The in vitro models include hydrogels that serve as platforms for the study of the blood–brain barrier and new methods for tumor treatment, limiting the damage of stroke and promoting the uptake of therapeutic agents into the central nervous system. To date, such experimental blood–brain barrier models have not been implanted or integrated in humans [93].

### 2.3. Astrocytes Derived from Stem Cells and Their Potential in Tissue Engineering

Astrocytes have important functions in normal and pathological states. Activated astrocytes are present in almost all neurological diseases. Most studies to date have been performed on animal experimental systems, mostly because of ease of access, maintenance in culture, and because of difficulty in obtaining primary human astrocytes. Due to interspecies differences, human astrocytes are preferred in experiments. In addition to the isolation of human astrocytes from various sources, as described above, induced pluripotent stem cells (iPSCs) have also been a focus of research. Almost all types of neural cells, including neurons, astrocytes, oligodendrocytes, neural stem cells, pericytes, and microglia, can be derived from iPSCs by considering developmental principles [105,106]. The iPSC-derived neural cells are a valuable tool for research, such as developing new therapeutic strategies, elucidation neurological disease mechanisms, and studying the physiology of the nervous system in health and disease [106,107].

**Figure 4:**
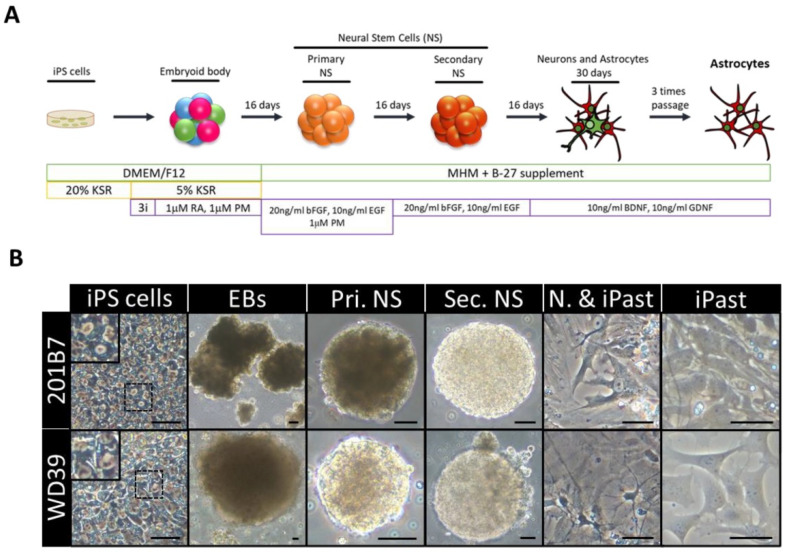
Induction of Human Astrocytes (iPasts). (**A**) Design of the human iPast induction protocol under serum free condition. Abbreviations: DMEM/F12: Dulbecco’s Modified Eagle Medium: Nutrient Mixture F-12; MHM: Media Hormone mix; B-27™: optimized serum-free supplement; KSR: KnockOut™ Serum Replacement; 3i: 3 µM CHIR99021, 3 µM 431542, 3 µM Dorsomorphine; RA: Retinoic acid; PM: Purmorphamine; bFGF (FGF-2): Basic fibroblast growth factor; EGF: Epidermal Growth Factor; BDNF: Brain-Derived Neurotrophic Factor; GDNF: Glial cell line-Derived Neurotrophic Factor. (**B**) Representative images of cells at each step of iPast induction process from the two iPSC control lines 201B7 and WD39. Abbreviations: EBs: Embryoid Bodies; Pri. NS: primary neurospheres; Sec. NS: secondary neurospheres; N. & iPasts: Neurons and iPasts; iPasts: human iPSC-derived astrocytes. The cells in the insets at iPSC stage are higher magnifications of cells in dashed boxes and indicative of good quality iPSCs with clear perinuclear halos. Scale bars: 50 µm (20 µm for iPast stage). [110].

The source of iPSCs are somatic cells that can be reprogrammed with transcription factors, such as SOX2, OCT4, KLF4, and MYC. This transformation technique has allowed the study of a variety of diseases and generated the concept of “disease in a dish”, which allows the modeling of disease phenotypes in a tissue culture dish [107,108]. The iPSCs are pluripotent like embryonic stem cells. They can be efficiently expanded and induced into all cell types in the human body under appropriate culture conditions. The iPSCs provide an unlimited source for subsequent differentiation into cell types of interest. Since they are reprogrammed from human somatic cells, concerns about species differences associated with animal models can be avoided. They also retain their original genomic features, such as chromosomal abnormalities and gene mutations. They remain intact after differentiation and can be used to study the effects of genomic defects on cellular functions, which is particularly valuable in drug development research (Figure 4) [107,109].

Human astrocytes derived from human iPSCs exhibit typical characteristics of physiological astrocytes and respond to various stimuli. Therefore, they are a suitable experimental model for studying astrocyte functions and reactivation under healthy and pathological conditions of the human nervous system [111]. Astrocyte differentiation techniques using human iPSCs are far from simple. They are lengthy and complex and the cells require the use of serum containing factors known to promote glial differentiation from neural precursor cells [112,113]. This is the reason why the isolation of astrocytes from human brain is still attractive and preferred by many laboratories over the iPSC technique. Compared to neurons, astrocytes are formed at a much later stage of embryonic development. This means that the differentiation process of astrocytes from iPSCs takes longer than that of neurons. Reports in the literature on the differentiation time of astrocytes from human iPSC vary and can last between 80 and 180 days [107,114,115].

The use of human iPSC-derived astrocytes in neurological disease modeling dates back to 2012. Early studies on neurodegenerative diseases were based on two-dimensional cell cultures. Although they provided important insights into brain dysfunction at a cellular level, their major limitation was that they did not allow for the proper spatial organization and developmental progression of cells in the brain. In contrast, neural cells derived from human iPSCs allow growth in cerebral organoids, which are self-organized, three-dimensional aggregates with cellular diversity and cytoarchitectures more similar to the human brain. They provide more sophisticated tissue architecture and microenvironmental signals than a traditional two-dimensional system [116,117,118]. Pathological changes in various neurodegenerative diseases are also reflected in astrocytes derived from human iPSCs. For example, iPSC-derived astrocytes in Alzheimer’s disease show a different morphology, with lower complexity and aberrant marker localization, compared to normal astrocytes [119]. The iPSC-derived astrocytes from patients with frontotemporal dementia affect neurons by inducing increased oxidative stress and transcriptional profile changes in previously healthy neurons [120]. Three-dimensional culture systems with hydrogel and iPSC-derived neurons and astrocytes are used to study Rett syndrome [107,121,122].

Stem cells have also been isolated from human exfoliated deciduous teeth (SHEDs) for use in neural differentiation studies in vitro [123]. These originate from the neural crest and are therefore particularly suitable for the induction of neural differentiation. A three-step protocol for neural differentiation of SHEDs cells was developed [123]. SHEDs treated according to this new differentiation protocol gave rise to mixed neuronal/glial cell cultures, opening up new possibilities for in vitro studies of neuronal and glial specification and expanding the potential for the use of such cells in experimental models and future treatment strategies [123].

## 3. Natural Biomaterials for Astrocyte Cell Models

### Hydrogels

Hydrogels are hydrophilic polymers commonly used in regenerative medicine and for reconstructive purposes [124]. They form a network that provides a suitable extracellular environment for cellular infiltration and a scaffold for cell ingrowth and matrix deposition. The hydrogel systems can also be used for drug delivery in in vitro systems, e.g., for the delivery of anti-inflammatory agents, including growth factors, corticosteroids, minocycline, broad-spectrum cell cycle inhibitors, and others [125,126].

Natural hydrogels are versatile compounds commonly used in cell culture research. They include collagen, hyaluronic acid, chitosan, alginate, fibrin, chitosan, and hydrogels derived from decellularized tissues [127]. The mechanical properties of the manufactured hydrogel scaffolds can be adjusted according to the tissue properties for which they are designed. For example, trauma to soft tissue can result in an irregular cavity, such as in muscles after wounding or even in the brain and spinal cord. Other properties of natural hydrogels include biodegradability and biocompatibility, low cytotoxicity, mimicking the physiological environment, and the ability to form the hydrogel into an injectable form and combine it with various therapeutic agents. Of course, spinal cord and brain injuries with tissue defects cannot heal in the same way and to the same extent as in other organs [128,129,130]. The advantageous properties of hydrogels include the ability to inject them into the lesion site, customize the lesion’s geometry, fill the cavity, release therapeutics over longer periods of time, and deliver cell-based therapies. Thus, injectable biomaterials conform to the cavity, unlike biomaterial matrices that are already manufactured and implanted into the tissue. In addition, injectable biomaterials in the liquid state have the advantage that they can be combined with growth factors or therapeutics before injection into the tissue, thus serving as drug carriers to promote regenerative processes in the tissue [127,131,132]. On the other hand, natural hydrogels also have some limitations, such as weak mechanical properties and batch-to-batch variability in manufacturing. Therefore, natural hydrogels are often combined with synthetic ones to produce composite polymers with improved properties [129,130,133].

**Figure 5:**
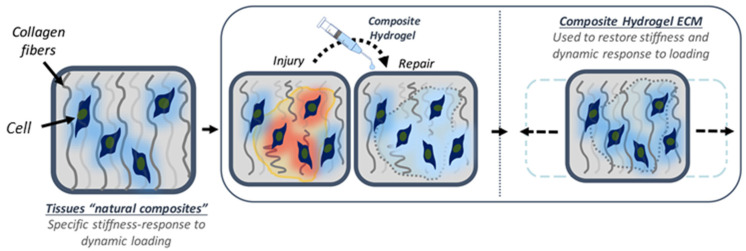
The use of collagen hydrogel for regeneration [135].

The growth characteristics of astrocytes in culture depend on the culture environment. Astrocytes cultured on two-dimensional coverslips or in simple monolayer tissue culture exhibit a diverse morphology, similar to the morphology of reactive cells found in vivo, such as in brain lesions [134]. Hydrogels can influence the growth pattern. In particular, collagen hydrogels, representing the three-dimensional environment, can more accurately model growth conditions in vivo (Figure 5).

They keep astrocytes in a quiescent state and enable them to grow in a three-dimensional environment that resembles their in vivo environment. This can be seen in the morphological features of the cells, which resemble those in the human brain and show a lower level of GFAP expression. Therefore, the use of three-dimensional hydrogels creates an in vitro environment that is more similar to the in vivo environment of astrocytes than two-dimensional monolayer cultures. The three-dimensional collagen hydrogels are particularly useful for testing astrocyte reactivity to potential stem cell therapies and for studying brain endothelial barrier function in vitro [136,137,138].

In addition, hydrogels can be stabilized against degradation, usually with hyaluronic acid. These hyaluronic acid-based hydrogels can be used to reduce unwanted scarring, in contrast to native hyaluronic acid, which induces astrocyte activation and proliferation [137,138]. In such systems, cells can grow with lower activation, which is more similar to their in vivo counterparts. Another source of hydrogels is fibrin, which can also significantly improve the regenerative environment by reducing astroglial scarring [139,140].

Hydrogels provide an important basis for the three-dimensional modeling of neural tissue. They are particularly useful in the study and experimental treatment of neurodegenerative diseases. They provide the opportunity to study combined cell interactions, including neurons and glia. Hydrogels used for three-dimensional models often involve complex macromolecules that form the extracellular matrix. For example, Matrigel is composed of proteins such as laminin and collagen, which are important for mechanical support and biochemical signaling [122]. In three-dimensional systems, more complex interactions can be modeled effectively. A critical obstacle in the development of primary three-dimensional neural tissue analogs is the need to support multiple cell types in the same environment [141]. To obtain a true three-dimensional model, the encapsulation of neural cells in hydrogels is an increasingly popular and important technique. In particular, with numerous cell types in such models, encapsulation in hydrogels requires the simultaneous incorporation of all cell types into the material. To successfully develop a primary three-dimensional cell model, it is necessary to understand the interactions of the cells with the hydrogel carrier. Since astrocytes, oligodendrocytes, neurons, and endothelial cells, which are often incorporated together, differ in their growth rate, it is important to prepare all cell cultures beforehand [142,143].

Besides natural hydrogels, synthetic hydrogels are also very interesting as an astrocyte cell model. Neurogel is a biocompatible poly(N-[2-hydroxypropyl]methacrylamide) hydrogel that reduces the reactive response of astroglia to injury by preventing scar formation, as measured by GFAP expression. Neurogel also promotes axonal growth into the matrix [144,145]. Moreover, neurons can functionally recover when grown on such biomaterials in co-culture with astrocytes [146].

## 4. Guidance Scaffolds

### 4.1. Electrospun Fibres Guidance Scaffolds

**Figure 6:**
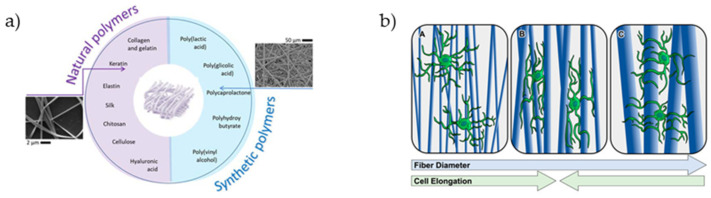
(**a**) Synthetic and natural polymers used for the fabrication of electrospun fibers [147]. (**b**) Effect of aligned fiber diameter on astrocyte elongation [32].

Electrospun fibers can be used for guidance scaffolds that direct the migration of astrocytes and the expansion of neurites of neurons in cell culture. Various biocompatible polymers such as polylactic-co-glycolic acid and poly-ε-caprolactone, polypropylene carbonate microfibers, or poly-L-lactic acid microfibers can be used (Figure 6).

Experiments with such materials implanted in a rat model with spinal cord injury showed that the astrocytes migrate into the channels along these fibers after several weeks of implantation, and axonal regeneration is localized in these areas of astrocyte migration. Electrospun collagen nanofibers promoted the sprouting of nerve fibers in injured animal models [148,149].

### 4.2. Topographical Guidance Scaffolds

**Figure 7:**
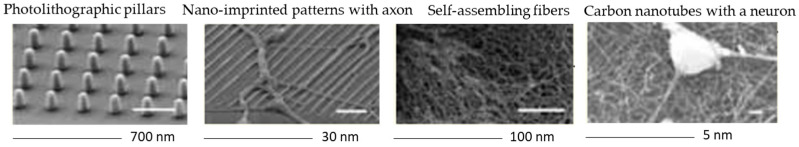
Different techniques for the preparation of topographical guidance scaffolds.

In the developing nervous system, and in some cases during regeneration, cells migrate along tracts of aligned extracellular matrix fibers and glial cells. The most prominent example may be the guidance of cortical neurons during histogenesis of the cerebral cortex, which are guided along cells of the radial glia [150,151]. In vitro, this can be simulated with fibrous materials with a suitable physical structure and chemical composition (Figure 7).

The fibrous material provides a physical scaffold for the growing cells and mimics the fibrous neural architecture and fibrous extracellular matrix structure in native tissue. The guidance scaffolds must provide appropriate mechanical support for cell growth and have favorable topographical properties for cell adhesion, proliferation, and differentiation [152]. Comparable to the injectable biomaterials described above, they can also be used as drug-delivery agents that slowly deliver drugs into the tissue (or a local area in an in vitro setting). Besides the general requirements for biomaterials used for cell experiments, such as biocompatibility, these materials must be non-mutagenic, nontoxic, and nonimmunogenic, as these properties can hinder cell growth in cell models [153]. Polymer fibers with sizes in the nanometer and micrometer range are used as topographical guidance scaffolds for tissue engineering applications. Like microgrooved surfaces, aligned fibers present anisotropic topography to cells, both in vitro and in vivo [151].

## 5. Ligand Patterned Surfaces

As the extracellular matrix is a complex environment that has a major impact on astrocyte growth and response, surface patterning is essential for studying manufacturing techniques and how this artificial environment can affect astrocyte response d. The extracellular matrix has a complicated geometry and is composed of numerous molecules. With the new techniques in bioengineering, it is possible to reproduce this complex environment, even for in vitro experiments [154]. Owing to this complexity, surface patterning is a useful in vitro technique to study the effect of artificial extracellular matrix structure on astrocyte growth and response. Porous surfaces have been developed to modify cellular growth and adhesion and to allow the transport of nutrients [155,156,157]. The surfaces may therefore influence astrocyte gene and protein expression, which depend on porosity, pore size, and thickness. It has been documented that astrocytes show variations in the expression of their important markers, such as glutamate transporter (GLAST), glial fibrillary acidic protein (GFAP), NG2 chondroitin sulphate proteoglycan (NG2), and glutamate transporter-1 (GLT-1), which depend on the structure of the extracellular matrix. The ability to mimic the topography of the extracellular matrix makes electrospun polymer fibers suitable for tissue engineering. With several biodegradable polymers as components, it is possible to regulate the fiber diameter and their orientation, which makes them particularly attractive for in vitro experiments on the nervous system [157,158].

Experiments with patterned substrates have shown that they control cell orientation and function. Extracellular matrix proteins patterned in various forms can influence the polarization of the internal organization of astrocytes. In addition, they influence the cell division axis. The observed elongated morphology of astrocytes could be manifested by the interaction of the ligand patterned surfaces when these cells grow on such substrates [159,160].

## 6. Conclusions

With the growing use of biomaterials in modern medicine, their potential uses and applications are also increasing. Due to the research and development of new biomaterials, it is not only possible to perform new experiments in the in vitro environment, but also to improve the properties of the developed biomaterials and discover new ones that can promote the growth and differentiation of cells in in vivo situations and hinder the processes of inflammation and degeneration. This is particularly important in neuroregenerative medicine, for the future treatment of spinal cord and brain injuries.
